# Transit and Metabolic Pathways of Quercetin in Tubular Cells: Involvement of Its Antioxidant Properties in the Kidney

**DOI:** 10.3390/antiox10060909

**Published:** 2021-06-03

**Authors:** Daniel Muñoz-Reyes, Ana I. Morales, Marta Prieto

**Affiliations:** 1 Toxicology Unit, University of Salamanca, 37007 Salamanca, Spain; danimr@usal.es (D.M.-R.); martapv@usal.es (M.P.); 2 Department of Physiology and Pharmacology, University of Salamanca, 37007 Salamanca, Spain; 3 Group of Translational Research on Renal and Cardiovascular Diseases (TRECARD), 37007 Salamanca, Spain; 4 Institute of Biomedical Research of Salamanca (IBSAL), 37007 Salamanca, Spain; 5 National Network for Kidney Research REDINREN, RD016/0009/0025, Instituto de Salud Carlos III, 28029 Madrid, Spain

**Keywords:** quercetin conjugates, renal intake, kidney metabolism, renal efflux, antioxidant, renoprotection

## Abstract

Quercetin is a flavonoid with antioxidant, antiviral, antimicrobial, and anti-inflammatory properties. Therefore, it has been postulated as a molecule with great therapeutic potential. The renoprotective capacity of quercetin against various toxins that produce oxidative stress, in both in vivo and in vitro models, has been shown. However, it is not clear whether quercetin itself or any of its metabolites are responsible for the protective effects on the kidney. Although the pharmacokinetics of quercetin have been widely studied and the complexity of its transit throughout the body is well known, the metabolic processes that occur in the kidney are less known. Because of that, the objective of this review was to delve into the molecular and cellular events triggered by quercetin and/or its metabolites in the tubular cells, which could explain some of the protective properties of this flavonoid against oxidative stress produced by toxin administration. Thus, the following are analyzed: (1) the transit of quercetin to the kidney; (2) the uptake mechanisms of quercetin and its metabolites from plasma to the tubular cells; (3) the metabolic processes triggered in those cells, which affect the accumulation of metabolites in the intracellular space; and (4) the efflux mechanisms of these compounds and their subsequent elimination through urine. Finally, it is discussed whether those processes that are mediated in the tubular cells and that give rise to different metabolites are related to the antioxidant and renoprotective properties observed after the administration of quercetin.

## 1. Introduction

Quercetin is a flavonoid present in the diet in high proportions. Its main sources are fruits, vegetables, and teas [1]. High concentrations of this flavonoid have been described in kale, onions, berries, apples, red grapes, black tea, etc. Incorporation of quercetin in different commercial food supplements is also common [2]. From a chemical point of view, the nomenclature for quercetin is 2-(3,4-dihydroxyphenyl)-3,5,7-trihydroxy-4H-chromen-4-one. It is a flavonol with a hydroxyl group in position 3, typical of flavonoids, and four additional hydroxyl groups, in positions 5, 7, 3′, and 4′ (Table 1) [3].

In nature, quercetin can be found free as quercetin aglycone or conjugated as a quercetin derivative, the latter being the predominant form. Among the most abundant quercetin derivatives in the diet are quercetin glycosides and ethers, while prenylated, sulfated, and glucuronide derivatives are found in smaller proportions (Table 1) [4]. The presence of different groups attached to quercetin changes the biochemical activity and bioavailability of the molecules when compared to aglycone [5]. Quercetin can also be found as dihydroquercetin (taxifolin) in nature, the reduced form of quercetin, which has two stereocenters on the C ring, as opposed to quercetin, which has none (Table 1) [6].

Quercetin glycosides are derivatives of the flavonoid where one or more hydroxyl groups have been replaced by different types of sugars, such as glucose, rhamnose, or rutinose. The main glycosylated forms of quercetin are O-glucosides, which are formed from an O-glycosidic bond between the hydroxyl group in position 3 of quercetin (Table 1) and the different carbohydrates. The presence of the carbohydrate increases the solubility of quercetin. More than 20 quercetin glycosides have been described; among the most common are quercetin 3-O-glucoside present in peas or sage, and quercetin 3-O-rhamnoglycoside (rutin) in cherries, spinach, or grapes [7].

Quercetin ethers are formed between a hydroxyl group on aglycone and an alcohol group on another molecule. These derivatives are very abundant, and contain sugars present in great abundance in nature as substituents. Among the most common quercetin monoethers are isorhamnetin (3’-methyl ether) or tamarixetin (4’-methyl ether), present in onions and honey [8,9].

Prenylated quercetin derivatives are less common than the glycosylated and ether derivates in natural sources. Structurally, in C-prenylflavonols, the prenyl groups are attached to the carbon atom of the flavonol backbone [10]. The addition of a prenyl group increases the hydrophobicity of aglycone and enhances the biological function of quercetin [11]. Examples of prenylated quercetin derivatives include 8-prenyl-quercetin in edible plants and 6,5’-di-C-prenylquercetin in Turkish mulberry [12,13].

In nature, there are also sulfated conjugates of quercetin, such as the 3,7,3’,4’-tetrasulfate derivative, isolated from the leaves of *Flaveria bidentis* [10] and also quercetin glucuronides such as quercetin 3-O-glucuronide, which is present in wines and medicinal plants such as *Hypericum hirsutum* and *Nelumbo nucifera* [14]. However, quercetin sulfates and glucuronides occur primarily as metabolites after quercetin biotransformation in the body (see Section 2.1).

Quercetin has strong antioxidant activity in vitro, and it is considered one of the most powerful ROS and RNS scavengers [7]. In addition to its antioxidant activity, it shows antiviral, antimicrobial, and anti-inflammatory properties [15]. Therefore, it has been postulated as a molecule with great therapeutic potential. Although the pharmacokinetics of quercetin have been widely studied and the complexity of its transit through the body is well known, the metabolic processes that occur in the kidney are less known. This review delves into the molecular and cellular events that trigger quercetin and/or its metabolites in the tubular cells, which could explain some of the protective properties of this flavonoid against oxidative stress produced by toxin administration. 

## 2. Quercetin Pharmacokinetics

### 2.1. Quercetin Absorption and Metabolism

The hydrolysis of quercetin glycosides occurs partially in saliva. It gives rise to quercetin aglycone in a deconjugation process, increasing the lipophilicity of the molecule. In this way, it can interact with the cellular lipid bilayer and cellular and subcellular components and tends to accumulate in cells. However, absorption from saliva is very low and tends to form quercetin-salivary protein aggregates. In the stomach, glycosides are stable, but the aglycone can undergo chemical degradation due to the strong acidic conditions of the gastric environment, resulting in phenolic acids such as protocatechic acid [16]. 

Quercetin derivatives are absorbed in the intestine depending on the radicals bound to quercetin aglycone [17]. The hydrolysis of quercetin glycosides occurs mainly in enterocytes via the action of lactase-florizin hydrolase (LPH) on the cell surface and/or intracellular β-glucosidases [7]. Glucuronide derivatives of quercetin in the diet are also hydrolyzed in enterocytes to form aglycone via lysosomal β-glucuronidases [18]. Regarding the prenylated, ether, and sulfated derivatives of quercetin, no intracellular hydrolysis mechanisms have been described.

Quercetin metabolism occurs mainly in the gastrointestinal tract [19]. In rats, about 93% of quercetin aglycone, or the one from hydrolyzed derivatives, is metabolized in the intestine after absorption. Efficient glucuronidation of quercetin occurs in enterocytes by UDP-glucuronyltransferases (UGT), methylation by catechol O-methyltransferases (COMT), and sulfation by sulfotransferases (SULT). In order to go from the cell to the bloodstream, a reconjugation process has to occur, and conjugated quercetin metabolites are formed. Therefore, quercetin aglycone is present in low amounts in plasma. The two main metabolites in humans that pass into the blood from the enterocyte are quercetin 3-O-glucuronide and quercetin 3’-O-sulfate, which are transported from the enterocyte to the liver through the portal vein [14,18]. So far, no enzymatic transformations of quercetin metabolites in plasma have been described.

**Figure 1:**
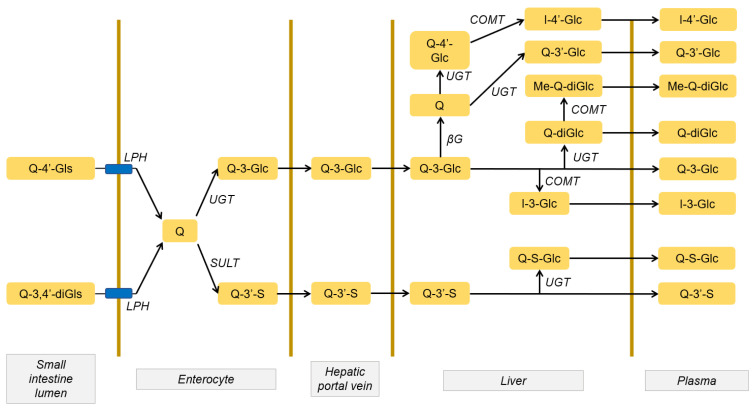
Metabolism of quercetin glycosides as they pass through the body. Quercetin glycosides are metabolized on their way through the enterocyte, and the metabolites are transported to the liver through the portal vein. Subsequently, metabolites quercetin 3-O-glucuronide and quercetin 3-O-sulfate undergo a second transformation in the liver before returning to the bloodstream. Modified figure from [20], adapted with permission from British Journal of Nutrition. Gls: glycoside, diGls: diglycoside, Glc: glucuronide, Q: quercetin, S: sulfate, SULT: sulfotransferase, UGT: glucuronyltransferase, GT: glucosyltransferase, I: isorhamnetin, COMT: methyltransferase, LPH: lactase-florizin hydrolase.

Although most quercetin is metabolized in the small intestine, the liver contains all the enzymatic systems that allow its complete metabolism through reactions of methylation, sulfation, and glucuronide conjugation. After hepatic metabolism, other metabolites are formed that enter the bloodstream. The main ones are quercetin diglucuronide, isorhamnetin 3-O-glucuronide, and quercetin sulfate monoglucuronide (Figure 1) [20].

It worth noting that there are differences in the metabolism of quercetin between different species. In humans, most quercetin methylation occurs in the liver, while sulfation takes place primarily in the enterocyte [16]. In contrast, in mice, there is greater activity of UGTs in the enterocyte [21].

### 2.2. Distribution and Accumulation of Quercetin and its Metabolites 

Once in the bloodstream, more than 80% of quercetin metabolites bind to plasma proteins, mainly albumin [7,22]. Therefore, around 20% of quercetin metabolites are in free form and can enter the tissues.

Quercetin and its metabolites tend to accumulate in the organs involved in its metabolism and excretion. Furthermore, it is suggested that quercetin and its metabolites could accumulate in cellular mitochondria [23]. Quercetin and its metabolites are distributed in many organs, such as the lungs, heart, and kidney [24]. As with many other xenobiotics, the concentrations of quercetin/metabolites reached in the kidney are usually high in relation to the administered dose [7]. This is because it is a highly irrigated organ, in addition to the main xenobiotic elimination organ. The antioxidant effects of quercetin on the kidney have been widely demonstrated [24,25]. The kinetic processes that take place in this organ could contribute to those beneficial effects, and that will be discussed below.

## 3. Transit of Quercetin through the Kidney 

The metabolites present in urine differ from those present in plasma and kidney tissue (Table 2). This is due to the fact that in the kidneys, there are processes of (a) filtration of the plasma in the glomerulus and discharge into the proximal tubules of the filtered plasma; (b) tubular reabsorption, in which a portion of the metabolites is reabsorbed by the tubular epithelial cell and is transported to the blood; and (c) tubular secretion, whereby the efflux of quercetin metabolites from blood into the urine occurs.

### 3.1. Quercetin Metabolite Uptake by Tubular Cells

**Figure 2:**
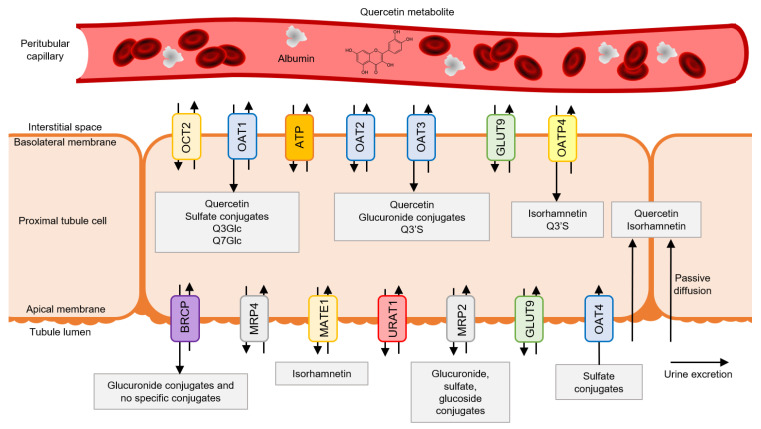
Quercetin transporters and their metabolites in proximal tubular cells. In summary, quercetin metabolites enter the tubular cell through passive diffusion of the influx transporters OAT1, OAT3, and OATP4 via tubular secretion and transporter OAT4 via reabsorption. The efflux transporters BCRP, MRP2, and MATE1 cause effective elimination of quercetin metabolites for urine excretion.

Glucuronide and sulfated conjugates of quercetin and isorhamnetin represent more than 90% of the total quercetin metabolites in plasma (see Section 2.1). Therefore, these are the metabolites that reach kidney tissue through systemic distribution [20]. In the proximal tubular cells, the entry of substances such as quercetin and its metabolites takes place mainly through influx transporters in the basolateral membrane and transporters in the apical membrane via tubular reabsorption (Figure 2) [26].

Quercetin metabolites undergo deconjugation processes to give rise to quercetin aglycone, which crosses the basolateral membrane mainly via passive diffusion due to its relative hydrophobicity [26]. Furthermore, quercetin uses active transport to enter the cell, as it has an affinity for organic anion transporters (OATs). In the basolateral membrane of the proximal tubular cells, there is a high expression of these transporters. Specifically, there are the isoforms OAT1, OAT2, and OAT3, which regulate the entry of organic anions from the bloodstream to the cells of the proximal tubular cells. Quercetin has an affinity for the OAT1 and OAT3 isoforms, which allows its entry into the tubular cell via active transport coupled to the efflux of α-ketoglutarate [27,28].

Sulfated conjugates, such as quercetin 3’-O-sulfate, have a high affinity for the OAT1 and organic anion transporting peptide-4 (OATP4) [26,29]. Quercetin 3’-O-sulfate is also transported by OAT3 but has a lower affinity for this isoform, while glucuronide conjugates, such as quercetin 3’-O-glucuronide, have a higher affinity for OAT3. In contrast, quercetin 3-O-glucuronide and quercetin 7-O-glucuronide are weak substrates of OAT1 and OAT3. This indicates that the position and type of substituent are decisive in the affinity of the metabolite for the transporter [26]. The transport of methylated conjugates such as isorhamnetin occurs through passive diffusion, given the planar configuration of the metabolite, and active transport mediated by organic anion transporting peptide (OATP), mainly through the OATP4 isoform, which is highly expressed in the basolateral membrane of proximal cells [30,31]. The presence of influx transporters for glycosylated quercetin metabolites in tubular cells has not been detected as no quercetin glycosides arise in the tubular cells.

Although tubular secretion has been addressed as the main mechanism by which quercetin metabolites access tubular cells, two different but related routes are also possible: glomerular filtration and tubular reabsorption, considering aglycone filtering. Hydrophilic metabolites in plasma filter into the glomerulus and pass into the tubules. Given the lipophilic nature of quercetin, the aglycone could also reach the tubular cell through tubular reabsorption in the apical membrane after glomerular filtration. Although no studies have been conducted to prove it, this hypothesis is reinforced by two facts: (a) glomerular filtration is a passive process, and aglycones can be reabsorbed in the apical membrane of proximal tubular cells; (b) aglycones are not found in urine, revealing that filtered aglycones must be reabsorbed and metabolize in the tubular cell [32].

Regarding the different metabolites, their possibility of reabsorption depends on their water solubility. Methylated metabolites, such as isorhamnetin, could also be reabsorbed in the apical membrane after glomerular filtration or deconjugation of a multiconjugated metabolite of isorhamnetin to give rise to quercetin aglycone (see Figure 2). For most hydrophilic metabolites, such as glucuronides and sulfates, passive diffusion is not contemplated, and therefore, it is more difficult for reabsorption to take place under physiological conditions. However, it is known that, unlike the other OAT transporters, the OAT4 transporter is expressed in the apical membrane of the proximal tubules, and the sulfated metabolites of quercetin have an affinity for this transporter [29]. Therefore, the OAT4 transporter could be involved in the reabsorption from urine to the tubular cell of sulfated metabolites, such as quercetin 3’-O-sulfate, since the reabsorption process is mediated via passive diffusion and transporters.

### 3.2. Quercetin Metabolism in Tubular Cells

The demethylation and deglucuronidation processes of quercetin conjugates can also take place in the kidney [33]. Tubular epithelial cells have high β-glucuronidase activity. This enzyme is responsible for the deconjugation of the main metabolites, such as glucuronides, to give rise to quercetin aglycones [34]. The aglycones present in the tubular cells could be due to the activity of this enzyme, since the kidney shows a high activity of the same, and furthermore, practically no quercetin aglycones are found in plasma (see Section 2) [35]. However, this deglucuronidation could be followed by instantaneous sulfation, and subsequent reglucuronidation, giving rise to the main metabolites found in the kidney, the glucuronide-quercetin sulfate conjugates [36]. 

The sulfotransferase 1A1 (SULT1A1) isoforms are expressed in the cytosol of renal cells, so they can be responsible for the quercetin sulfation [37]. In tubular cells, there are different isoforms of UGTs, which produce glucuronidation of quercetin. Of the 19 isoforms described in the literature, 4 isoforms are significantly expressed in the kidney [38]. The UGT1A9 isoform is expressed at high levels in the human kidney [39]. In addition to UGT1A9, there are other isoforms, although they have lower levels of expression, such as UGT1A7 and UGT1A8. However, the expression of these last two isoforms is relatively abundant. They are responsible for the glucuronidation of flavanols such as quercetin in tubular cells. The UGT1A7 isoform performs more efficient glucuronidation than the UGT1A8 isoform [40].

Renal cells also show COMT activity [41]. These enzymes carry out efficient methylation of quercetin aglycones to form methylated derivatives in kidney cells. The main methylated metabolites of quercetin in kidney cells are 3’-O-methylquercetin (isorhamnetin) and 4’-O-methylquercetin (tamarixetin) [36]. Methylquercetin conjugates have also been found in tubular cells, such as glucuronided conjugates (isorhamnetin 3-glucuronide and isorhamnetin 4’-glucuronide) [20].

The kidneys also possess β-glycosyltransferase (GT) activity that allows the formation of glycosylated metabolites from quercetin aglycones present in tubular cells and, therefore, facilitates their excretion through the urine [36].

Therefore, the tubular cells present all the enzymatic machinery to carry out a third biotransformation of quercetin in the kidney. The metabolic conversions that occur include a complex combination of metabolite deconjugation followed by immediate sulfation, glucuronidation, methylation, and glycosylation [19,20].

### 3.3. Quercetin Metabolite Accumulation in Tubular Cells

As in plasma, quercetin is found in conjugated form in tissues, suggesting that quercetin’s biological activity could be due to its metabolite activity [19]. In the kidney, more than 90% of quercetin is in its conjugated form due to the enzymatic activity of tubular cells (see previous section) [19]. Diglucuronide, monoglucuronide, and glucuronide-sulfated metabolites of quercetin have been found in kidney tissue [42]. Furthermore, isorhamnetin is another of the most abundant metabolites in humans [43]. It is also noteworthy that quercetin glycoside metabolites have not been found in blood, which means that glycosides taken in the diet undergo complete biotransformation before reaching the tubular cells [44]. However, quercetin glycosides are found in the tubular cell due to the metabolism that takes place in the latter.

Several studies have shown that oral administration of quercetin derivatives results in higher levels of quercetin metabolites in the kidney than administration of quercetin aglycone because of greater bioavailability and easier access to the tubular cell (see Section 2.1 and Section 3.1). These include isoquercetin and prenylated derivatives. Oral administration of isoquercetin produces an increase in the accumulation of quercetin in the kidney (397%) and methylquercetin (318%) compared to oral administration of quercetin aglycone [45]. A similar effect is observed with the administration of prenylated quercetin derivatives, since a 385% increase in quercetin accumulation and a 736% increase in methylquercetin are observed [35].

In the kidneys of rats, after chronic feeding of a diet rich in quercetin for 6 weeks, the presence of a high amount of methylquercetin monoglucuronide sulfate was observed, a multiconjugated metabolite that represented 65% of the total quercetin metabolites present in the homogenate. This metabolite was also the predominant form in the plasma of rats, formed after methylation, glucuronidation, and further sulfation of quercetin [19]. In another study on pigs, two experiments were performed to determine the accumulation of quercetin metabolites in different tissues after a single dose and repeated doses over 4 weeks of quercetin. In the repeated dose experiment for 4 weeks, a greater accumulation of quercetin and methylated metabolites was observed in the kidney (two times higher than in the liver) [36]. A higher proportion of methylated metabolites is observed in kidney tissues of humans and rats, which appear almost exclusively conjugated with glucuronide and sulfate [43]. The large amount of glucuronide metabolites in kidney tissues stands out [33]. In pig kidney tissues, the proportion of methylated metabolites is lower, the proportion of aglycones being higher in pigs [36].

Accumulation of quercetin metabolites in tubular cells suggests that biological effects may occur in the kidney. Therefore, the accumulation of pharmacologically active metabolites should be enhanced as a therapeutic strategy.

### 3.4. Quercetin Metabolites’ Luminal Efflux by Tubular Cells 

Many drugs and their conjugated metabolites are excreted in the urine via tubular secretion. As already mentioned above, efflux transport in proximal tubular cells takes place at the apical membrane. The apical membrane mainly expresses the transporters MRP2/4 (multidrug resistance-associated protein 2/4), MDR1 or glycoprotein-p (multidrug resistance protein 1), BCRP (breast cancer resistance protein), MATE1 (multidrug and toxin extrusion protein 1), and OAT4. These proteins transport the flavonoid conjugates from the proximal tubular cells to the urine, finally excreting them [46]. The BCRP transporter is not a specific transporter, and quercetin, isorhamnetin, and quercetin 3’-sulfate can also cross the apical membrane via tubular secretion through this transporter [47].

Glucuronide conjugates of quercetin are transported mainly by MRP2 and BCRP, while sulfated metabolites such as quercetin 3’-O-sulfate are transported by MRP2 and OAT4 [26,29]. Methylated metabolites such as isorhamnetin have an affinity for MATE1, which acts as a competitive inhibitor of the protein in the presence of other drugs. Therefore, the consumption of foods rich in quercetin with other drugs could generate drug interactions at this level [48]. Glycosylated metabolites formed in tubular cells also have an affinity for the MRP2 transporter [49]. No influx transporters of glycosylated quercetin conjugates have been detected (see Section 3.1). In contrast, efflux transporters of these are expressed in the apical membrane. Therefore, these metabolites may have been formed in the tubular cells through the renal metabolism of quercetin.

### 3.5. Quercetin Metabolites in Urine 

**Figure 3:**
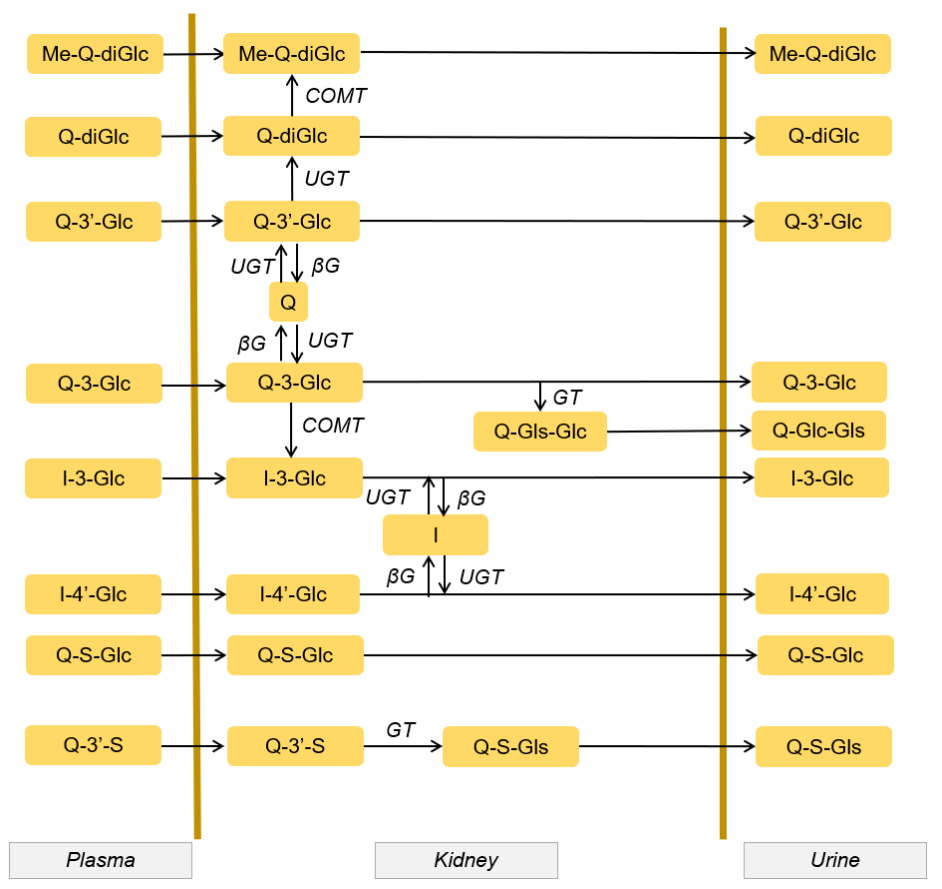
Hypothetical scheme of quercetin metabolism in the tubular cell. The metabolites present in plasma reach the tubular cell and undergo enzymatic transformations that increase their solubility and allow them to be excreted through the urine. Modified figure from [20], adapted with permission from British Journal of Nutrition. Gls: glucoside, Glc: glucuronide, Q: quercetin, S: sulfate, I: isorhamnetin, UGT: glucuronyltransferase, GT: glucosyltransferase, COMT: methyltransferase, βG: β-glucuronidase.

In humans, the excretion of quercetin metabolites through the urine represents between 20 and 60% of the total quercetin intake [26], while a large amount of the metabolites are eliminated through the lungs and feces [3].The main metabolites of quercetin present in human urine are quercetin monoglucuronide sulfates, methylated quercetin monoglucuronides, and quercetin diglucuronide. There are differences between metabolites in human plasma and urine. For example, quercetin 3’-sulfate is a major metabolite in plasma, but small amounts of the conjugate are absent or found in urine. This is due to renal metabolism, which allows the conversion of aglycone and quercetin metabolites (see Figure 3, Section 3.2) [20].

## 4. Antioxidant and Renoprotective Effects of Quercetin and Its Metabolites 

### 4.1. Quercetin Antioxidant Activity 

Quercetin has a wide variety of pharmacological properties, most notably its antiobesity, antiviral, antimicrobial, anti-inflammatory, and cardioprotective effects [15]. Recently, it has also been reported to have anti-COVID-19 activity and is therefore postulated as a promising therapy for the treatment of disease caused by SARS-CoV2 [50]. Numerous studies attribute these properties to the antioxidant action of quercetin, becoming the focus of food and pharmaceutical industries [7].

Oxidative stress is the consequence of an imbalance between the production of oxidizing substances and the cellular antioxidant mechanisms. It plays a central role in numerous biological processes, including aging, neurodegenerative, respiratory, and cardiovascular diseases, and kidney damage [51]. Reactive oxygen species (ROS) and reactive nitrogen species (RNS) are involved in the initiation and progression of kidney pathologies, including diabetic nephropathy, hypertension, ischemia-reperfusion injury (IRI), etc. ROS and RNS are generated in both renal tubules and vascular cells during cellular and tissue stress. The main sources of oxidative stress in the kidney come from NADPH oxidase and the mitochondrial respiratory chain. Low ROS and RNS levels are essential to maintain the optimal redox state. However, cell and tissue stress create an imbalance between ROS and RNS production and elimination, and therefore, antioxidant activity is reduced, producing a cluster of these species and causing cell damage and tissue dysfunction [52]. In this context, antioxidant substances such as quercetin can act as scavengers and neutralize ROS and RNS, maintaining the cellular redox state.

Chemically, quercetin’s antioxidative activity is due to a catechol group in the B ring, a 2,3-double bond in conjugation with a 4-oxo function in the C ring, and a hydroxyl group at positions 3 and 7 in heterocyclic ring (see Table 1) [7]. It has strong antioxidant activity in vitro and is considered one of the most powerful ROS and RNS scavengers [7]. However, it exerts its antioxidant activity by regulating glutathione levels and modulating the activity of antioxidant enzymes and different signaling pathways [53].

Some studies indicate that quercetin conjugates have lower antioxidant activity than aglycone. Its antioxidant capacity could be arranged in the following order: aglycone > tamarixetin = isorhamnetin> quercetin 3-O-glucuronide > isorhamnetin 3-O-glucoside. This indicates that the hydroxyl group at position 3 of quercetin plays an important role in the antioxidant activity of the flavonoid [54]. However, a study carried out by Lesjak et al. (2018) [55] showed that methylated metabolites of quercetin, such as isorhamnetin and tamarixetin, exhibited greater antioxidant activity than quercetin in reducing abnormal lipid peroxidation [55]. In another study, quercetin 3-glucuronide was shown to be more effective than aglycone in protecting against oxidative stress given its greater chemical stability [56]. Therefore, it is not clear whether aglycone or the metabolites have the highest antioxidant activity. These metabolic transformations probably produce changes in their physical–chemical properties and cause the conjugates to reach the kidney in larger or smaller concentrations. 

#### 4.1.1. Scavenger Activity

Quercetin acts as a reducing agent by inhibiting or reducing free radical toxicity. The main ROS produced from molecular oxygen in cells are the superoxide anion, perhydroxyl radical, hydrogen peroxide, and hydroxyl radical [53]. Several studies show the excellent scavenging capacity against ROS of quercetin and some of its metabolites, such as isorhamnetin [57,58].

#### 4.1.2. Glutathione Level Regulation

Several studies reveal that quercetin modulates intracellular glutathione production, increasing the concentration of reduced glutathione [24,59]. Glutathione is a tripeptide that, in its reduced form (GSH), breaks down hydrogen peroxide in water as it is oxidized (GSSG), maintaining the cellular reducing environment and acting as a scavenger [60].

#### 4.1.3. Antioxidant Enzyme Modulation 

Under physiological conditions, an efficient antioxidant system buffers the oxidative action of ROS, minimizing intracellular oxidative damage. Antioxidant enzymes catalyze the conversion of ROS to oxygen and water [51]. The main enzymes responsible for the defense mechanism against oxidative stress are superoxide dismutase (SOD), catalase (CAT), and glutathione peroxidase (GPx). In this sense, numerous studies have revealed that quercetin restores the cellular redox state by enhancing the expression levels of these enzymes [61,62].

#### 4.1.4. Antioxidant Signaling Pathways 

**Figure 4:**
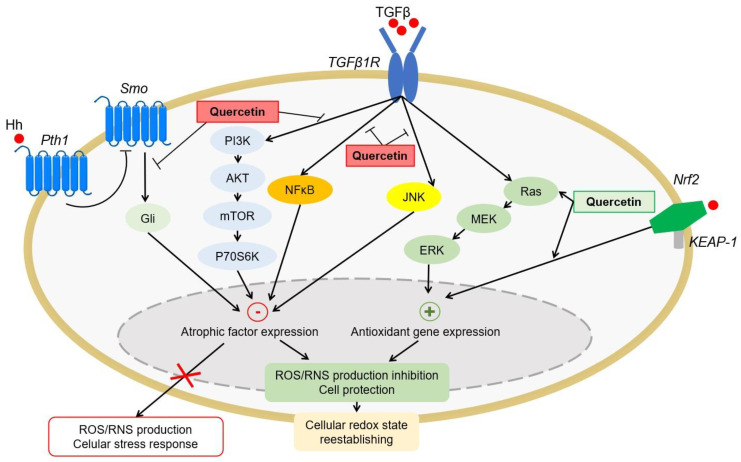
Effect of quercetin on the regulation of molecular mechanisms induced by oxidative stress. Quercetin inhibits the Hedgehog, PI3K/AKT, JNK, and NF-κB signaling pathways. Furthermore, quercetin induces the MAP kinases and Nrf2 signaling pathways. As a consequence, there is an increase in the expression of antioxidant enzymes and the production of ROS is inhibited, reestablishing the cellular redox state. +: indicates induction; -: indicates inhibition.

Oxidative stress also causes dysregulation of cellular signaling pathways that maintain cellular homeostasis. Some quercetin and derivatives have the ability to modulate these routes to reestablish the optimal redox state of the body and repair the damage caused by the induction or inhibition of these routes. Quercetin exerts its antioxidant effect by positively regulating the signaling pathways of nuclear factor 2 related to erythroid 2 (Nrf2) [63] and mitogen-activated protein kinases (MAP kinases) [24] and negatively regulates the phosphatidylinositol 3-kinase (PI3K/Akt) [64], activated B-cell nuclear factor kappa light chain enhancer pathways (NF-κB) [65], and Hedgehog [66] (Figure 4). Specifically, Nrf2 is the main transcription factor that modulates the antioxidant response, regulating a wide variety of antioxidant enzymes and resistance to oxidative stress. Quercetin and some of its derivatives, such as hyperoxide or taxifolin, have the ability to positively regulate its activation [63,67,68].

### 4.2. Renoprotective Activity of Quercetin and Its Metabolites 

As previously described, after the ingestion of foods rich in quercetin, or the administration of the flavonoid, quercetin and its derivatives are metabolized, and a wide distribution occurs, mainly to the most irrigated tissues and those involved in their excretion. For this reason, the kidney is one of the organs that presents a greater accumulation of quercetin and its metabolites in the body and is, therefore, one of the main targets of its antioxidant effects. Although the concentration of quercetin and/or its metabolites in the kidney is relatively low in relation to the administered dose, they appear to exert pharmacological effects on renal cells [24]. Numerous studies have shown that quercetin derivatives have renoprotective activity based on their antioxidant activity [24,25]. In our laboratory, numerous studies have been carried out that have shown that part of the nephroprotective effect of quercetin against numerous nephrotoxic agents is due to its antioxidant activity. These nephrotoxic agents include cadmium [69], cisplatin [70], and contrast agents [71].

Most in vivo studies have attributed to quercetin aglycone or quercetin glycosides the execution of therapeutic effects on different target organs, such as the kidney [24,25], without taking into account the extensive metabolism that occurs on the flavonoid, since they are compounds that are administered in vivo. In contrast, the large accumulation of quercetin and its conjugates in the kidney suggests that the biological effects are probably due to the action of metabolites in their transit through the kidney cell. 

Some in vitro studies indicate that quercetin’s antioxidant activity may be due to some of its metabolites. Thus, in a study with HK-2 cells, i.e., human proximal tubular cells, it was suggested that quercetin is metabolized within a few minutes or even seconds of being incorporated into the culture medium. These cells contain UGT enzymes so it is possible that quercetin is transformed when cells are incubated with the flavonoid [72]. Furthermore, it has been suggested that these metabolites could be involved in the induction of antioxidant defense mechanisms through antioxidant response elements (AREs), inducing the expression of antioxidant enzymes [72]. Another in vitro study revealed that taxifolin has renoprotective effects [63]. Taxifolin modulates the Nrf2 signaling pathway, favoring the translocation of the transcription factor Nrf2 to the nucleus, where it binds to AREs and activates the transcription of genes encoding phase II antioxidant and detoxifying enzymes [73]. Among these enzymes is UGT, which favors the formation of quercetin glucuronides (see Section 3.2). Consequently, the quercetin conjugates themselves that access the kidney are inducers of their own metabolism and favor the formation of glucuronide conjugates. Therefore, according to these studies, glucuronide metabolites could be responsible for antioxidant and renoprotective activity and not quercetin itself, at least in part.

#### Aspects to Be Elucidated and Limitations of Current Studies

A relevant aspect to elucidate is whether the nephroprotective effect of quercetin (or quercetin derivate) is due to its metabolism in the kidney or in other organs. As already mentioned (see Section 3.2), the tubular cell has all the necessary machinery to carry out the metabolic conversions of quercetin, suggesting that the nephroprotective effect is due to renal metabolism and the accumulation of pharmacologically active metabolites.

Another consideration would be whether the renoprotection and/or antioxidant activity depends solely on the transit of a metabolite through the kidney or its accumulation in the kidney is necessary. One possibility would be that the effect could be a mixture of both processes. The transit of metabolites as they pass through the tubular cell can induce different signaling pathways, such as the Nrf2 pathway, without the need to accumulate, and quercetin would act as an autoinducer of its metabolism [73], enhancing the antioxidant activity of metabolites. However, a low efficiency of efflux transporters could be favoring the accumulation of metabolites in the cell and, thus, the antioxidant effect. However, the type of quercetin derivative and the administered dose should also be considered, since this determines the derivative that accesses the renal cell and its physicochemical properties, as shown in this review.

Current knowledge presents numerous limitations to answering these questions: a) there are no quantification studies of the enzymatic activity of the enzymes that catalyze the formation of quercetin metabolites in the tubular cell; b) there are not enough accumulation studies of the different metabolites in renal cells; and c) quercetin accumulation is related to antioxidant and/or renoprotective activity, but not to the formation of metabolites or their transit through the tubular cell. Studies in humans allow us to determine the biological effects of quercetin derivatives, but they do not allow us to determine in real time the transit or accumulation of flavonoids in tissues such as the kidney, liver, or brain with the techniques currently available. In vitro studies seem promising for this type of study, although they have limitations since they are isolated systems that may not reflect the processes that occur in vivo, such as the transit of metabolites between cells and their distribution between different organs. 

The possibility of clarifying these aspects would allow us to establish strategies such as the encapsulation of an active metabolite to favor its accumulation in the kidney. In our laboratory, it has been shown that the encapsulation of quercetin with Pluronic F127 enhances the biopharmaceutical properties, increases the bioavailability of the flavonoid, and maintains the renoprotective properties against quercetin aglycone [74]. Similarly, the encapsulation of the active metabolite would facilitate its access to the tubular cell, its renal metabolism, and its accumulation. However, all the limitations that studies currently have should be taken into account, so perhaps the key lies in the combination of in vivo and in vitro studies to elucidate the renoprotective mechanisms and design strategies with clinical potential.

## 5. Conclusions 

Quercetin metabolites that enter the kidney through the blood undergo conversion reactions due to renal metabolism to give rise to more water-soluble metabolites. Although knowledge of the mechanism of action of quercetin metabolites is still scarce, the transit and/or accumulation of these metabolites in the renal cell suggests that the antioxidant and/or renoprotective effects can be due to them, at least in part, and not to the aglycone itself, through the modulation of intracellular and molecular events. Favoring the accumulation of a pharmacologically active quercetin metabolite would enhance the antioxidant and/or renoprotective effect. This would allow the development of new strategies for the treatment of oxidative stress produced by the administration of nephrotoxins.

## Figures and Tables

**Table 1:**
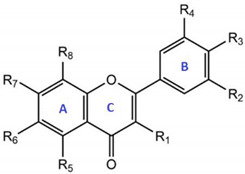
Main derivatives and metabolites of quercetin.

Quercetin Derivatives		Radicals							
		R1	R2	R3	R4	R5	R6	R7	R8
Aglycone	Quercetin	OH	H	OH	OH	OH	H	OH	H
Reduced aglycone	Dihydroquercetin (taxifolin)	(OH)^1^	H	OH	OH	OH	H	OH	H
Glycosides	Quercetin 3-O-rhamnoside (quercitrin)	O-Rham^2^	H	OH	OH	OH	H	OH	H
	Quercetin 7-O-rhamnoside (Vincetoxicoside B)	OH	H	OH	OH	OH	H	O-Rham	H
	Quercetin 3-O-rhamnoglucoside (rutin)	O-RG^3^	H	OH	OH	OH	H	OH	H
	Quercetin 3-O-glucoside (isoquercetin)	O-Gls^4^	H	OH	OH	OH	H	OH	H
	Quercetin 3-O-galactoside (hyperoside)	O-Gal^5^	H	OH	OH	OH	H	OH	H
Ethers	Quercetin 3′-methylether (isorhamnetin)	OH	H	OH	O-Met^6^	OH	H	OH	H
	Quercetin 4′-methylether (tamarixetin)	OH	H	O-Met	OH	OH	H	OH	H
	Quercetin 7-methylether (rhamnetin)	OH	H	OH	OH	OH	H	O-Met	H
Prenylated	8-prenylquercetin	OH	H	OH	OH	OH	H	OH	Pren^7^
	6,5′-di-C-prenylquercetin	OH	H	OH	Pren	OH	Pren	OH	H
Sulfated	Quercetin 3,7,3′,4′-tetrasulfate	OSO_3_^-8^	H	OSO_3_^-^	OSO_3_^-^	OH	H	OSO_3_^-^	H
	Quercetin 3-O-sulfate	OSO_3_^-^	H	OH	OH	OH	H	OH	H
	Quercetin 3′-O-sulfate	OH	H	OH	OSO_3_^-^	OH	H	OH	H
	Quercetin 4′-O-sulfate	OH	H	OSO_3_^-^	OH	OH	H	OH	H
	Quercetin 7-O-sulfate	OH	H	OH	OH	OH	H	OSO_3_^-^	H
Glucuronides	Quercetin 3-O-glucuronide	O-Glc^9^	H	OH	OH	OH	H	OH	H
	Quercetin 3′-O-glucuronide	OH	H	OH	O-Glc	OH	H	OH	H
	Quercetin 4′-O-glucuronide	OH	H	O-Glc	OH	OH	H	OH	H
	Quercetin 7-O-glucuronide	OH	H	OH	OH	OH	H	O-Glc	H
	Quercetin diglucuronide	O-Glc	H	OH	OH	OH	H	O-Glc	H

^1^ () indicates stereocenter; ^2^ Rham: rhamnose; ^3^ RG: rhamnosylglucose; ^4^ Gls: glucose; ^5^ Gal: galactoside; ^6^ Met: methyl; ^7^ Pren: 3-methyl-2-buten-1-il; ^8^ OSO3-: sulfate; ^9^ Glc: glucuronide acid.

**Table 2:** Percentage of quercetin metabolites localized in human plasma and urine after an enriched diet of quercetin derivatives. Data from [20].

Quercetin Conjugates (%)	Data from [20]	
	Plasma	Urine
Quercetin monoglucuronide	27.6	21.4
Quercetin diglucuronide	4.6	17.2
Methylated quercetin monoglucuronide	8.8	19.3
Methylated quercetin diglucuronide	-	11.1
Quercetin glucoside monoglucuronide	-	1.3
Quercetin glucoside sulfate	-	9.4
Quercetin monoglucuronide sulfate	10.5	20.3
Quercetin 3′-sulfate	48.5	Trace
